# Multidimensional Quantification of Macular Cone Activity in Pattern Electroretinography Using Discrete Wavelet Transform

**DOI:** 10.1167/tvst.14.9.17

**Published:** 2025-09-12

**Authors:** Yousif J. Shwetar, Melissa A. Haendel

**Affiliations:** 1 Joint Department of Biomedical Engineering, University of North Carolina and North Carolina State University, Chapel Hill, NC, USA; 2 Department of Genetics, University of North Carolina, Chapel Hill, NC, USA

**Keywords:** signal processing, wavelet transform, electroretinography, diagnostics, metrics

## Abstract

**Purpose:**

To evaluate discrete wavelet transform (DWT) features as quantitative biomarkers of macular cone function from pattern electroretinography (PERG) in macular-predominant inherited retinal diseases (mpIRDs).

**Methods:**

In total, 486 PERG recordings from 123 participants were obtained from the PERG–Institute of Applied Ophthalmobiology open-access data set and analyzed. Twenty mother wavelets were screened with an energy-to-entropy ratio criterion; six (haar, sym2, sym4, db4, coif1, fk4) were retained for feature generation. After feature cleaning and correlation pruning, a final set of 141 features was obtained and averaged per participant to avoid visit bias. Group separation was assessed with nonparametric statistics. Inverse-DWT signal reconstruction was performed with the sym2 wavelet to algorithmically determine time-frequency indices needed to preserve N35, P50, and N95 peaks. The smallest set of indices that achieved this was retained.

**Results:**

Sym2-D6-2 (38–75 ms, 13–27 Hz) emerged as the top discriminative feature (*r_es_* = 0.644, common-language effect size = 0.875) and correlated strongly with the clinical macular cone marker |P50–N35| (*r_corr_* = 0.95) across 67 normal participants (262 recordings). Compared with |P50–N35|, the same index showed tighter, nonoverlapping group distributions, a higher diagnostic area under the curve (0.875 vs. 0.835), and a larger effect size (*r_es_* = 0.644 vs. 0.576).

**Conclusions:**

DWT-derived time-frequency features, particularly sym2-D6-2, provide robust, multidimensional biomarkers of macular cone function. These quantitative endpoints hold promise for monitoring disease progression and evaluating therapeutics in mpIRDs.

**Translational Relevance:**

Sym2-D6-2 provides an objective metric of macular cone function that could serve as a quantitative endpoint in mpIRD trials.

## Introduction

Electroretinography (ERG) is a noninvasive test that records the retina's electrical response to controlled visual stimuli (flash, pattern reversal, etc.).^1^^,^^2^ Within the family of ERG techniques, pattern ERG (PERG) is especially valuable for assessing macular cone and retinal ganglion cell (RGC) function^3^^,^^4^; the PERG waveform is characterized by a positive inflection at ∼50 ms (P50), representing macular cone response, and a negative deflection at ∼95 ms (N95), representing RGC response.^5^ As a result, PERG has played a role in tracking the early loss of macular photoreceptors in select inherited retinal diseases (IRDs).^6^^–^^9^

IRDs encompass a broad group of genetic disorders that together affect roughly 1 in 2000 to 3000 individuals worldwide.^10^ Among them, cone-rod dystrophy (CRD), macular dystrophy (MD), and Stargardt disease (STGD) primarily damage macular cone photoreceptors, leading to central-vision impairment while sparing peripheral rods in early stages.^11^^,^^12^ Because PERG is most sensitive to macular cone activity, it is well suited for monitoring functional decline in these macular-predominant IRDs (mpIRDs).^13^ Prior studies show that STGD and MD often exhibit attenuated PERG amplitudes despite normal full-field electroretinograms (ffERGs),^9^^,^^14^^–^^16^ whereas CRD frequently presents with a selective reduction in P50 amplitude that tracks early cone loss.^8^^,^^17^^,^^18^

Conventional time-domain analysis of PERG provides useful summary metrics (e.g., P50 and N95 amplitudes, implicit times), but these traditional methods are one-dimensional and can be difficult to obtain. Time-frequency approaches, such as the wavelet transform (WT), offer distinct advantages by capturing both temporal and spectral characteristics, while scaling basis functions, enabling optimal multiresolution analysis. Of the WT methods, discrete WT (DWT) is simple and efficient, offering direct quantitative metrics for analysis. Previous work in the field of visual electrophysiology has demonstrated these time-frequency methods to be superior to traditional time-domain methods^19^^–^^26^: Sarossy et al.^27^ showed that adding DWT- and matching-pursuit–derived coefficients to conventional ERG amplitude markers nearly doubled the variance explained when predicting RGC counts in glaucoma eyes (*R*² increasing from 0.34 to 0.63), compared with amplitude markers alone. Hassankarimi et al.^28^ similarly demonstrated that an energy-based feature, the level 7 negative peak (7N) using an eighth-order Daubechies mother wavelet, successfully delineated early primary open-angle glaucoma from healthy controls even when N95 amplitudes overlapped. Gauvin et al.^29^ used DWT energy features at 20 Hz and 40 Hz to isolate the ON- and OFF-pathway contributions to the human ffERG, underscoring how carefully selected wavelet features can expose pathway-specific dysfunction.

**Figure 1.**
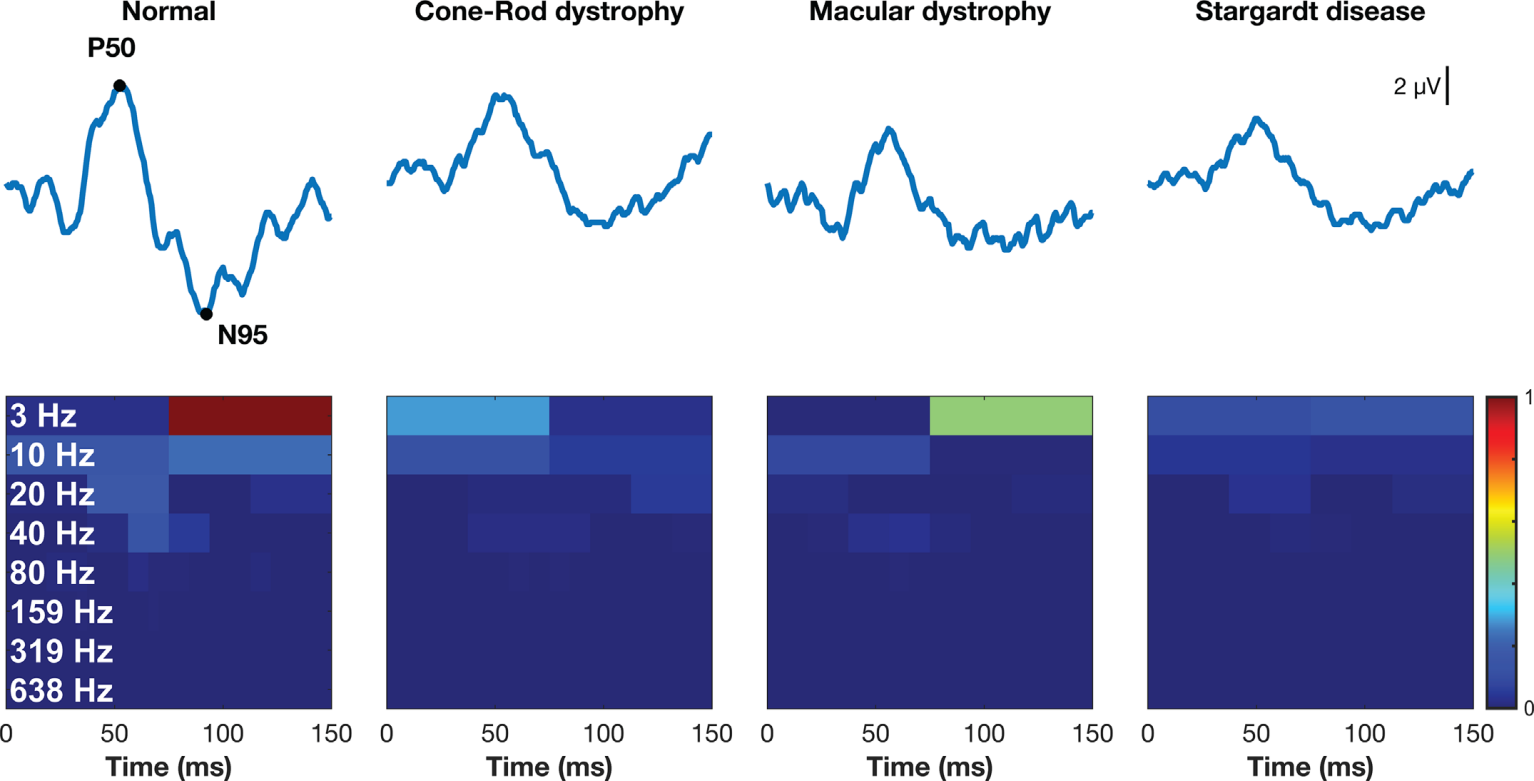
Representative PERG waveforms and their Haar-wavelet energy maps (scalograms) across diagnoses. *Top row*: Time-domain PERG traces from a healthy control (normal) and patients with CRD, MD, and STGD. Canonical peaks are annotated on the normal trace (P50, N95); *vertical scale bar* = 2 µV. *Bottom row*: Corresponding scalograms produced with the Haar mother wavelet. Color denotes relative energy in each index. In the normal eye, the bulk of energy is concentrated in the low-frequency A7 band (∼3 Hz) and in central-time indices drawn from D6 (∼20 Hz) and D7 (∼10 Hz), aligning with the P50 time window. In contrast, pathologic eyes show attenuated energy across these same indices, reflecting the loss of macular cone–driven activity.

While previous research highlights the advantages of DWT analysis in visual electrophysiology, a systematic evaluation of its application to PERG signals, specifically to capture macular cone dysfunction, remains unexplored. As illustrated in Figure 1, conventional time-domain markers can have amplitudes that overlap those of normal recordings, making subgroup discrimination using temporal markers far more difficult than with DWT-derived indices. Given this gap, the present study leverages the two-dimensional, multiresolution ability of the DWT to address three specific aims:

1. Select an optimal set of mother wavelets using energy-to-entropy, ensuring DWT analysis represents true physiological signal response and low noise. This will serve as a prerequisite for robust time-frequency analysis.
2. Derive time-frequency energy indices that represent canonical PERG time-domain markers like N35, P50, and N95. We are particularly interested in identifying time-frequency markers that temporally align with the macular cone response of the PERG (N35 → P50). This would be indicative of macular cone activity, or the lack thereof, as we expect to observe large differences in this response between normal recordings and those with mpIRDs.
3. Identify a minimal subset of wavelet coefficients that can reconstruct PERG waveforms and preserve N35, P50, and N95. This third aim will algorithmically reinforce our statistically driven second aim, while also obtaining an optimal set of wavelet coefficients for effective signal reconstruction.

To our knowledge, this is the first study to utilize time-frequency analysis to derive novel time-frequency features to enhance the assessment of macular cone integrity and combine all three aims in the assessment of PERG recordings in a single study. Ultimately, these refined, multidimensional biomarkers may provide standardized quantitative endpoints valuable for monitoring disease progression and assessing future therapeutic strategies.

## Methods

### Data Characteristics

PERG recordings were obtained from the open-access PERG–Institute of Applied Ophthalmobiology (IOBA) data set.^30^^,^^31^ The data set contains 1354 PERG recordings obtained across 336 clinical visits from 304 individuals at IOBA, University of Valladolid (Valladolid, Spain), between 2003 and 2022. Each record provides at least one signal from each eye along with age, sex, logarithm of the minimum angle of resolution (logMAR) visual acuity (VA), and up to three coexisting clinical labels assigned to the same participant spanning 69 etiologies. Many participants have a single entry, while others have related macular codes (e.g., macular dystrophy, central areolar choroidal dystrophy clinical labels for a single participant). Ethical approval for the original collection and informed consent were obtained; therefore, the present secondary analysis of anonymized data required no further review.

The PERG-IOBA data set already incorporates sweep-level artifact control: any epoch that exceeds 8 µV in fast-rejection mode or 50 µV in slow-rejection mode is discarded and replaced by interpolation, and at least 100 clean sweeps are averaged to produce each trace. We also restricted the cohort to records classified as normal or mpIRD (CRD, MD, or STGD). Note that we did not include normal participants who had mercury poisoning (120 recordings) or recordings without a VA measure (38 recordings). Cohort characteristics, including sex, age, and right eye (RE) and left eye (LE) VA, were summarized separately for each diagnostic subgroup. To maintain independent samples, only the first visit per participant was retained. As a result, recordings from eight IDs were excluded from analysis, as this was a patient on their second visit. The final analytic cohort consisted of 123 total participants providing 486 recordings, detailed fully in Table 1. Further details regarding the process for creating our cohort, its final combination of diagnoses (diagnosis1, diagnosis2, and diagnosis3), and commentary regarding their potential impact in interpretation are available in the supplemental material.

**Table 1.** Cohort Overview by Diagnostic Group

Diagnosis	No. of Participants (Male/Female)	Recordings	Age, Mean ± SD, Y	VA, RE/LE, Mean ± SD, logMAR
Normal	67 (23/44)	262	27.3 ± 17.7	0.20 ± 0.41/0.22 ± 0.42
Cone-rod dystrophy	12 (5/7)	46	32.2 ± 15.9	0.61 ± 0.45/0.55 ± 0.40
Macular dystrophy	29 (13/16)	114	41.0 ± 17.1	0.41 ± 0.37/0.40 ± 0.36
Stargardt disease	15 (6/9)	64	36.2 ± 15.9	0.57 ± 0.77/0.57 ± 0.56

Recordings were acquired in accordance with the International Society for Clinical Electrophysiology of Vision (ISCEV) standards for clinical PERG.^32^ Signals were acquired binocularly with a gold-foil corneal electrode (reference at the ipsilateral outer canthus, forehead ground). Participants fixated a 1.0° black-and-white checkerboard reversing at 4 reversals per second (rps) on a cathode-ray tube monitor subtending 12° × 16° at 1 m (mean luminance 45–50 cd m^–^^2^, near-maximal contrast). Data were digitized at 1700 Hz, hardware-filtered 1 to 100 Hz, and 150-ms analysis windows were averaged per ISCEV standards. Each averaged trace was linearly detrended using MATLAB’s (MathWorks, Natick, MA, USA) detrend function, which subtracts a zero-order polynomial without fitting a slope (DC offset). The final data point was then repeated so that every trace contains exactly 256 samples to achieve dyadic length.

### Discrete Wavelet Transform

Our primary aim was to translate the canonical time-domain peaks (N35, P50, and N95) into quantitative time-frequency features that can be compared across diagnoses. We applied the DWT, which recursively filters the signal into approximation (A) and detail (D) coefficients at dyadic scales. Each coefficient index is squared, yielding energy heatmaps (scalograms) that present the overall time-frequency energy of the signal. The maximum admissible decomposition level for every wavelet was determined automatically by MATLAB's wmaxlev function. The only exception to this was for the haar wavelet, where the maximum decomposition level was set to 7, as this brackets the 150-ms signal into two features representing P50 (0–75 ms) and N95 (75–150 ms) separately. Additionally, we used MATLAB's “per” (periodic) extension mode, which treats each 256-sample trace as circular and wraps the signal end-to-start during filtering. Periodic extension mode was preferred, as it enabled dyadic decomposition, which was ideal for temporal alignment of energy indices with key physiological responses. Additionally, PERG is recorded during continuous pattern reversal; wrapping the traces approximates continuity between successive cycles better than zero- or mirror-padding. Figure 1 presents an example of time-domain recordings, as well as associated DWT scalograms using the haar wavelet from a healthy control and from individuals with mpIRDs. Associated frequency ranges and center frequencies for each decomposition level are listed in Supplementary Table S2. Information regarding wavelet transform in ERG can be found here.^33^

### Energy and Entropy Calculation

To ensure robust spectral analysis, we computed the energy (*E*) and Shannon entropy (*H*) for each candidate wavelet:

$$\begin{array}{c} E=\sum_{i=1}^{N}{c_{i}}^{2} \end{array}$$

where *c_i_* are the wavelet coefficients for a specific decomposition level. Energy, here *E*, refers to the power of the signal within a given time-frequency bin, as quantified by squared wavelet coefficients. *H* measures the distribution of coefficients (and thus energies), done by normalizing them into probabilities (*p_i_*):

$$\begin{array}{c} p_{i}=\frac{{c_{i}}^{2}}{\sum_{j=1}^{N}{c_{i}}^{2}}=\frac{{c_{i}}^{2}}{E} \end{array}$$

and then applying the following:

$$\begin{array}{c} H\;=\;-\sum_{i=i}^{N}p_{i}\cdot log\left(p_{i}\right) \end{array}$$

Entropy measures the distribution of the energies, where values widely spread have high entropy, while tightly clustered energy produces low entropy. These quantities are useful as they provide complementary perspectives on how a particular wavelet captures and represents a signal. High energy suggests the wavelet decomposition has successfully captured a substantial portion of the signal's strength, while low entropy indicates decomposition is more ordered. To systematically evaluate candidate wavelets across our final PERG data set, the energy-to-entropy ratio for each wavelet was obtained and then averaged across all participants within each wavelet type as

$$\begin{array}{c} E\cdot H^{-1} \end{array}$$

For each wavelet, we computed the mean energy-to-entropy ratio across the full analytic cohort and derived 95% bootstrap confidence intervals (CIs) (10,000 participant-level resamples) to estimate statistical variability.^27^ This calculation was repeated separately for each retained decomposition level (D4, D5, D6, A5, A6) to check whether any wavelet performed uniquely well within a specific frequency window. Wavelets with high average ratios and narrow CIs were deemed optimal as they routinely demonstrated higher signal power and low disorder. Additionally, individual mother wavelets displayed structural attributes like compact support, near-linear phase, specific number of vanishing moments, or a combination of these features, making them particularly adept at resolving transient, sharply bounded components characteristic of the PERG waveform. Given these considerations, we first retained all mother wavelets that yielded the highest mean energy-to-entropy ratio in each decomposition-level analysis. Then, any wavelet family that was not included was hand-picked and included in the analysis, as we wanted to ensure we were able to evaluate the unique spectral characteristics of each wavelet family. Only wavelets that could be directly implemented in the DWT in MATLAB (as evidenced by the waveinfo function) and yielded three or more decomposition levels <100 Hz were implemented, as mother wavelets that yielded any fewer levels would negate the multidimensional advantage DWT offers. This ultimately limited energy-to-entropy analysis to 20 mother wavelets: haar, db2–db8, sym2–sym8, coif1–2, fk4/6/8.

### Statistical Analysis and Feature Reduction

We first extracted all wavelet indices (coefficients and energies) up to the maximum decomposition level for each mother wavelet (with the exception of haar, as previously stated). Given that these data had been passed through a 1- to 100-Hz bandpass filter, we removed detail levels D1 to D3, as these only contained frequency content >100 Hz. We also only retained the maximal approximation level, as this did not contain any redundant frequency content that the remaining detail levels contained. Recordings were then averaged within participants to form a single feature vector for each participant to account for repeated measures. Group differences were assessed with the Mann–Whitney *U* test. False discovery rate was controlled with the Benjamini–Hochberg procedure (*α* = 0.05). We also quantified effect sizes using the following two methods:

• Effect size (*r_es_*), defined as $r_{es}\;=\;\frac{Z}{\sqrt{N}}$, where *Z* is the Mann–Whitney *z*-statistic, and *N* is the total sample size.
• Common-language effect size (CLES) is the probability that a randomly chosen normal observation will exceed a randomly chosen mpIRD observation.^34^ It is represented as $CLES\;=\;\frac{U}{n_{Normal}\;\cdot \;n_{mpIRD}}$, with *U* being the Mann–Whitney *U* statistic and *n* being the group sample size. Values range from 0.5 (no discrimination) to 1.0 (perfect separation). For example, a CLES of 0.92 would mean that in 92% of all normal and mpIRD recordings, the normal PERG measure is higher.

To limit redundancy, we also assessed Spearman rho (|*ρ*|) between each feature. If two features exhibited a |*ρ*| > 0.9, the feature with the lower effect size *r_es_* was removed, as it provided less discriminatory power for the same information. This removed 51 features and created a final set of 141 features for statistical testing.

To directly benchmark our highest-performing features with the canonical time-domain marker of macular cone activity (|P50–N35|), we performed a three-part participant-level analysis. First, Tukey boxplots visualized group distribution for selected metrics. Second, we assessed diagnostic accuracy with a receiver operating characteristic (ROC) area under the curve (AUC) metric. Lastly, we calculated the Mann–Whitney effect-size *r_es_* to conduct direct comparison of discriminatory strength.

### Minimal Algorithmic Signal Reconstruction

We carried out a data-driven approach that identified the smallest set of wavelet indices that could faithfully reproduce each canonical PERG peak. Consistent with prior studies by Gauvin et al.,^26^ we selected the sym2 wavelet for minimal algorithmic signal reconstruction due to its advantageous properties (symmetric shape, near-linear phase response, and minimal phase distortion). For every trace, we generated a one-index inverse DWT from all coefficients in the D4, D5, D6, or A6 (<100 Hz) decomposition levels. Peak amplitudes and implicit times were measured in both the original signal and these reconstructed indices, and the mean absolute error (MAE) between these two was measured. The index with the lowest weighted MAE (µV × ms) across the entire normal cohort was retained for each peak. We then assessed the impact of adding another one of the high-performing indices with the top performing one—if including this additional index lowered the weighted MAE by 40% or more, it was retained. To relate these indices to conventional metrics, participant-level Pearson correlations (*r_corr_*) were calculated between each retained energy index value and the representative macular cone (|P50–N35|) and RGC (|N95–P50|) amplitudes.

## Results

**Figure 2.**
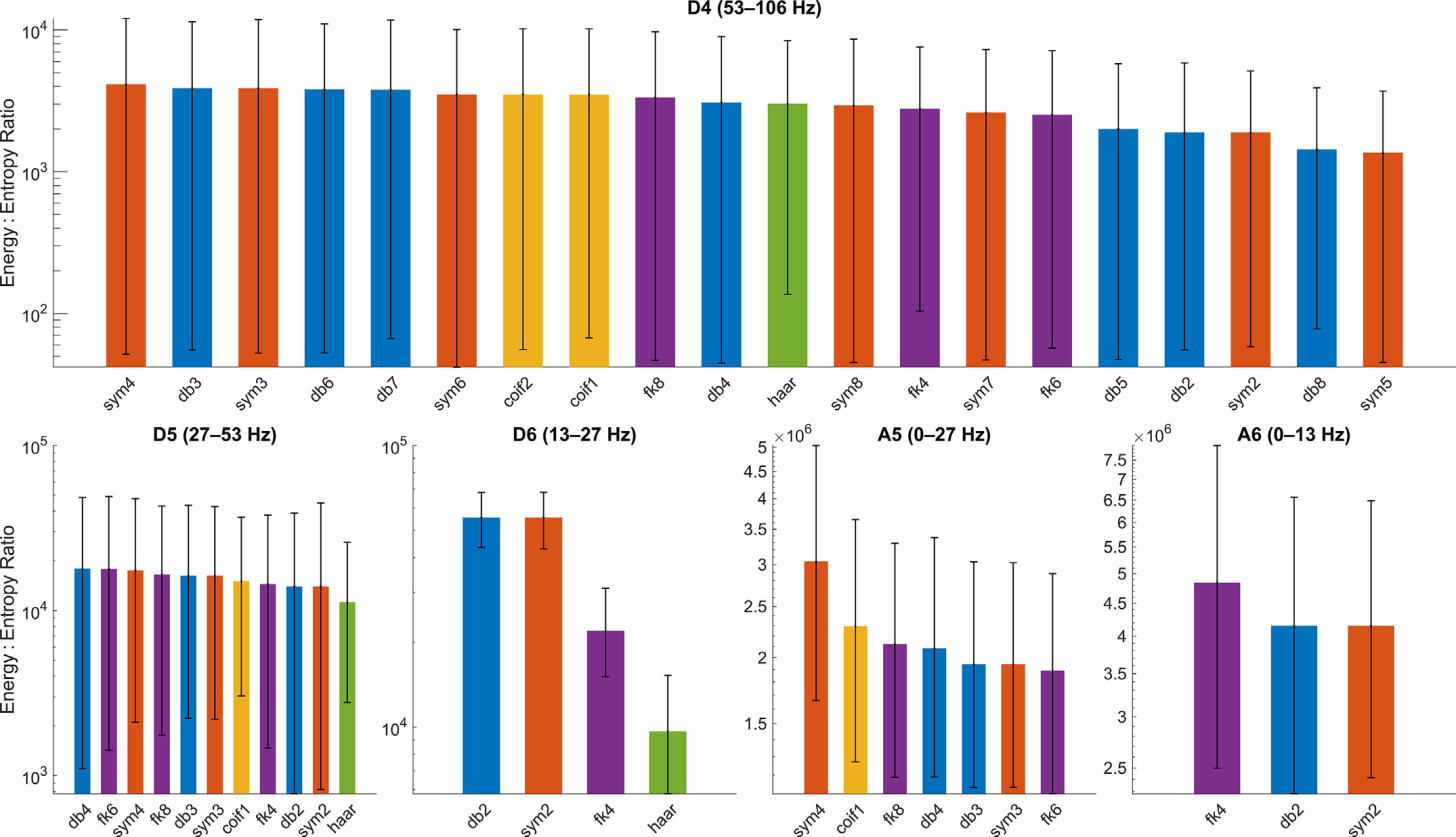
Decomposition-level energy-to-entropy screening of candidate mother wavelets. Mean energy-to-entropy ratios (E/H, y-axis on logarithmic scale) with 95% bootstrap confidence intervals (*n* = 10,000 recording-level resamples) are shown for all 20 wavelet families, stratified by the five physiologically relevant subbands (D4, D5, D6, A5, A6) across the analytic cohort (486 PERG recordings, 123 participants). Bar colors denote wavelet families: *orange* = Symlets (sym), *blue* = Daubechies (db), *yellow-gold* = Coiflets (coif), *purple* = Fejér–Korovkin (fk), *green* = Haar. The highest mean energy-to-entropy ratio in each decomposition-level analysis was retained for feature generation: D4–sym4, D5–db4, D6–sym2, A5–sym4, A6–fk4. Haar was the only metric to record a D7 and A7 and was thus retained. Coif1 was added to ensure representation of every family. At D6, db2 and sym2 were statistically indistinguishable (mean E/H ≈ 55,489; 95% CIs overlapped by >99%). Only sym2 was retained for its symmetric, near-linear phase response that minimizes phase distortion and has been routinely utilized in prior ERG work.

Figure 2 presents the energy-to-entropy ratios for every mother wavelet, stratified by five decomposition levels (D4, D5, D6, A5, and A6). We did not plot D7 and A7, as haar was the only wavelet to have a decomposition level 7 and was automatically included. For each panel, we retained the mother wavelet with the highest mean energy-to-entropy ratio: D4–sym4, D5–db4, D6–db2 and sym2, A5: sym4, A6: fk4. We also manually selected coif1 as we wanted to ensure we had a mother wavelet from each family represented in our analysis. For decomposition-level D6, db2 and sym2 are statistically indistinguishable (both with a mean energy-to-entropy ratio of 55,489.4 and 95% CIs overlapping by 99%). However, we opted for sym2 due to its symmetric, near-linear phase design, which minimizes phase distortion. Furthermore, we want to stay consistent with well-established previous work that has utilized sym2 for the same reasons.^26^ After feature reduction, statistical testing revealed the top-performing features based on effect sizes *r_es_* and CLES.

Sym2-D6-2 (38–75 ms, 13–27 Hz) emerged as the single most discriminative feature, with *r_es_* = 0.644 and CLES = 0.875, likely capturing P50. Other highly ranked features include coif1–D5-3 (38–56 ms, 27–53 Hz), which also aligned with the proximal component of P50 (*r_es_* = 0.621; CLES = 0.861), while sym2–D6-3 (75–112 ms, 13–27 Hz) was the top feature temporally aligned with N95 (*r_es_* = 0.581; CLES = 0.838). A complete summary of the top-ranked features that yield an effect size greater than that of |P50–N35| (*r_es_* = 0.576) is provided in Table 2 (all *P* < 0.001). We also conducted an eye-specific analysis to ensure our findings remained robust. When the data were repartitioned by recording side, sym2–D6-2 remained the top-ranked feature in LE recordings (*r_es_* = 0.606, CLES = 0.853); see Supplementary Table S2. When grouped by RE or “worst eye” (based on higher logMAR VA or smaller |P50–N35| amplitude), this modestly altered the lead feature (Supplementary Tables S3–S5). Full statistical details across all sensitivity scenarios are provided in the Supplementary Data.

**Table 2.** Top Four Discriminative Wavelet Coefficients (Ranked by Effect Size *r_es_*) Distinguishing Controls From mpIRD

Feature	Effect Size *r_es_*	CLES	Time Range (ms)	Frequency Band (Hz)
sym2–D6-2	0.644	0.875	38–75	13–27
coif1–D5-3	0.621	0.861	38–56	27–53
fk4–D6-2	0.586	0.841	38–75	13–27
sym2–D6-3	0.581	0.838	75–112	13–27

All Mann–Whitney *U* tests remained significant after Benjamini–Hochberg correction (*P* < 0.001 for every feature here).

**Figure 3.**
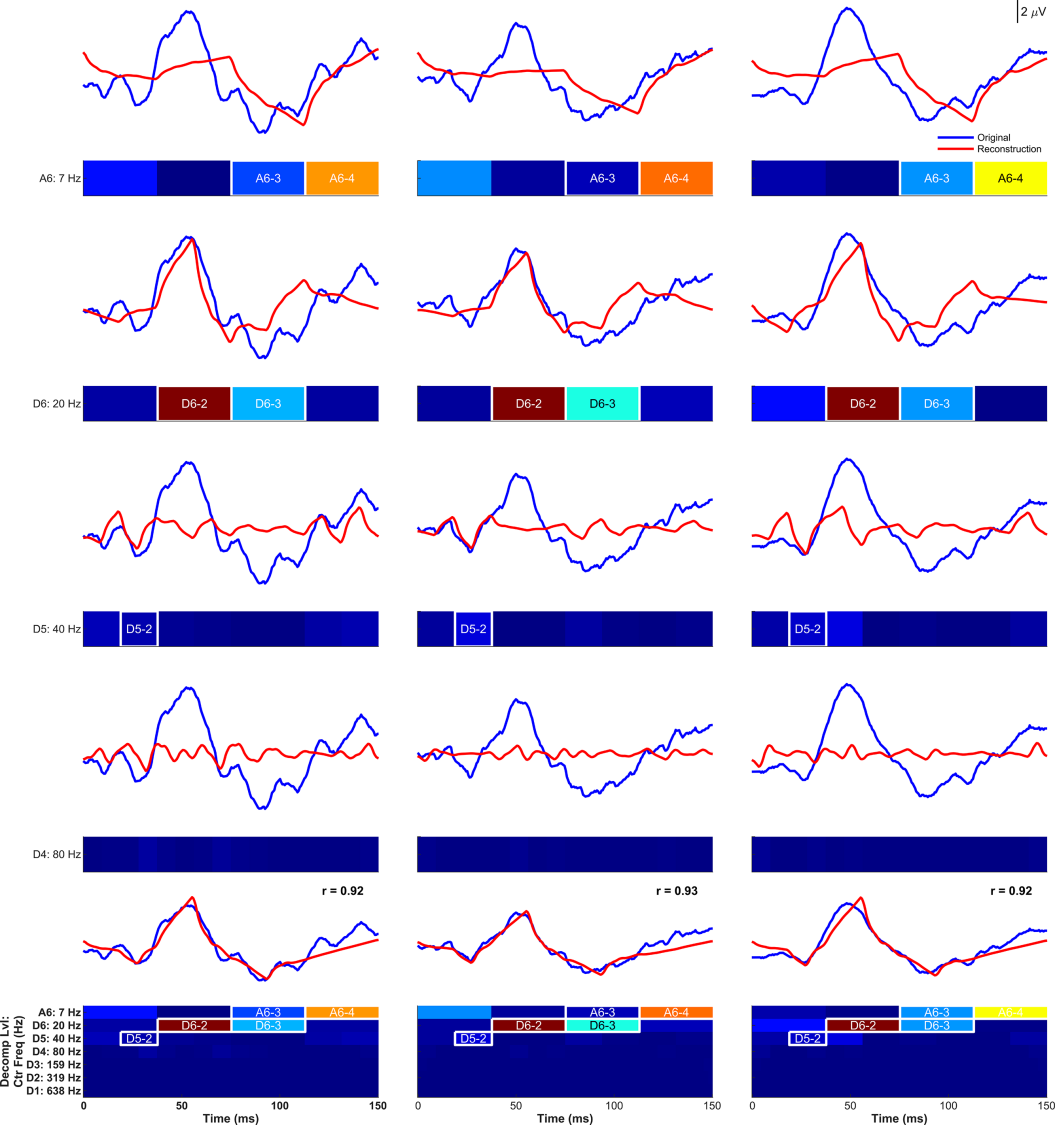
Algorithmic inverse-DWT reconstructions of a representative PERG trace using a minimal set of sym2 wavelet coefficients. Columns depict three separate normal recordings. Rows 1 to 4 show signals reconstructed using a singular band to assess which portions of the PERG waveform lie in each frequency band. For example, row 1 is reconstructed using only coefficients from level A6. *White boxes* highlight indices from a row that were algorithmically found to optimally resolve canonical peaks. This process is continued for rows 2 (D6), 3 (D5), and 4 (D4). Row 5 demonstrates reconstructed signals using only the identified indices from the previous four rows. Pearson correlation coefficients (*r_corr_* > 0.9) confirm high fidelity while preserving the canonical N35, P50, and N95 peaks, using only five indices. Ctr Freq, center frequency; Decomp Lvl, decomposition level.

Across all normal recordings, the top five indices that minimized the weighted amplitude-implicit time MAE for each canonical peak are provided in Table 3. The top-performing single indices for each canonical time component include sym2–D5-2 for N35 (2.17 µV·ms), sym2–D6-2 for P50 (6.01 µV·ms), and sym2–D6-3 for N95 (5.42 µV·ms). Upon further evaluation of Table 3, we found that sym2–D6-3 yielded low temporal MAEs and high amplitude MAEs, while sym2–A6-4 exhibited low-amplitude MAEs and high temporal MAEs. We attempted reconstructing these two metrics together and found both amplitude and temporal resolutions to improve markedly (from 5.42 to 3.25 µV·ms) and thus utilized this combined metric, sym2–D6-3 + sym2–A6-4, for N95. Similarly, for sym2–D6-2, we attempted reconstruction with a combination of each of the top two through five performing indices for P50, as shown in Table 3, and found the combination of sym2–D6-2 + sym2–A6-3 to yield a much lower weighted MAE (6.01 to 2.03 µV·ms) than any other combination. These signal reconstructions can be graphically appreciated in Figure 3, where full signals in blue are overlaid with inverse-DWT wavelet reconstructions in red, along with white box outlines in the associated scalograms. The bottom row overlays a trace rebuilt solely from the five selected indices, with canonical N35, P50, and N95 amplitudes and timings largely preserved. Note that no additional index for N35 lowered the weighted MAE by at least 40%.

**Table 3.** Error Metrics for Candidate sym2 Coefficients Used in Inverse-DWT Reconstruction of the Canonical PERG Peaks (N35, P50, N95)

Canonical Marker	Energy Index	Voltage MAE (µV)	Time MAE (ms)	Weighted MAE (µV × ms)
N35	D5-2	0.85	2.52	2.15
	D5-1	1.39	2.78	3.87
	D4-2	1.48	3.19	4.73
	D6-4	1.48	3.19	4.74
	D4-3	1.31	3.84	5.04
P50	D6-2, A6-3	0.48	4.77	2.31
	D6-2	1.09	5.53	6.01
	D4-6	2.90	3.23	9.38
	D5-3	2.81	3.69	10.36
	A6-3	2.30	7.37	16.99
	A6-2	3.41	5.36	18.27
N95	D6-3, A6-4	0.57	5.66	3.25
	D6-3	0.99	5.50	5.42
	A6-4	0.51	15.41	7.81
	D4-11	1.90	6.37	12.10
	D5-6	1.68	8.01	13.47
	D4-10	1.90	7.51	14.25

MAE, Mean Absolute Error.

**Figure 4.**
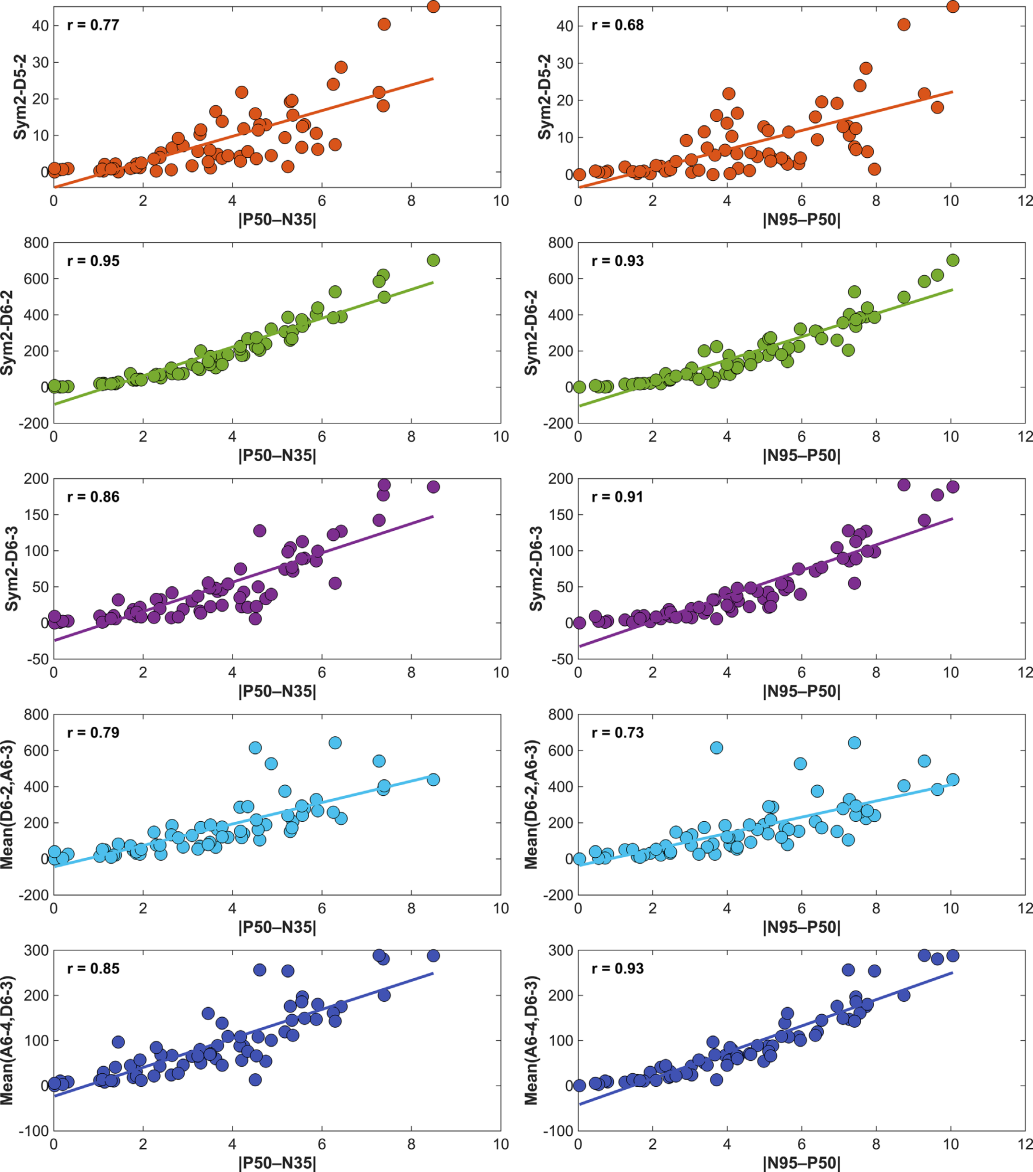
Correlation between sym2 wavelet-energy indices and canonical PERG amplitude metrics in normal participants. *Left column*: participant-level mean energies of sym2–D5-2, sym2–D6-2, sym2–D6-3, mean of sym2–D6-2 with sym2–A6-3, and mean of sym2–D6-3 with sym2–A6-4 plotted against the macular cone marker |P50–N35|. *Right column*: the same indices plotted against the RGC marker |N95–P50|. *Solid lines* represent least squares fits; Pearson correlation coefficients (*r_corr_*) are indicated in each panel. Sym2–D6-2 shows exceptionally strong associations with both |P50–N35| (*r_corr_* = 0.95) and |N95–P50| (*r_corr_* = 0.93), while Sym2–D6-3 is highly correlated with |N95–P50| (*r_corr_* = 0.91). Each point represents the average of all recordings from a single participant (*n* = 65). Two participants yielded three recordings with sym2–D5-2 values >4 SD above the mean and were thus omitted.

Figure 4 shows the within‐participant averaged linear correlations between these sym2 wavelet indices and the traditional temporal markers |P50–N35| and |N95–P50| in normal participants only. The resulting scatterplots revealed exceptionally strong linear correlations between sym2–D6-2 vs. |P50–N35| (*r_corr_* = 0.95) and vs. |N95–P50| (*r_corr_* = 0.93), as well as sym2–D6-3 vs. |N95–P50| (*r_corr_* = 0.91), with remaining comparisons available in Figure 4. We combined sym2–D6-2 and sym2–A6-3 for the P50 metric, as well as sym2–D6-3 and sym2–A6-4 for the N95 metric, using their mean values rather than analyzing these indices individually, as the two approximation indices were only included in conjunction with the detailed indices, respectively. Note that we omitted three recordings from two participants whose sym2–D5-2 values were four standard deviations greater than the mean of the remaining normal participants.

**Figure 5.**
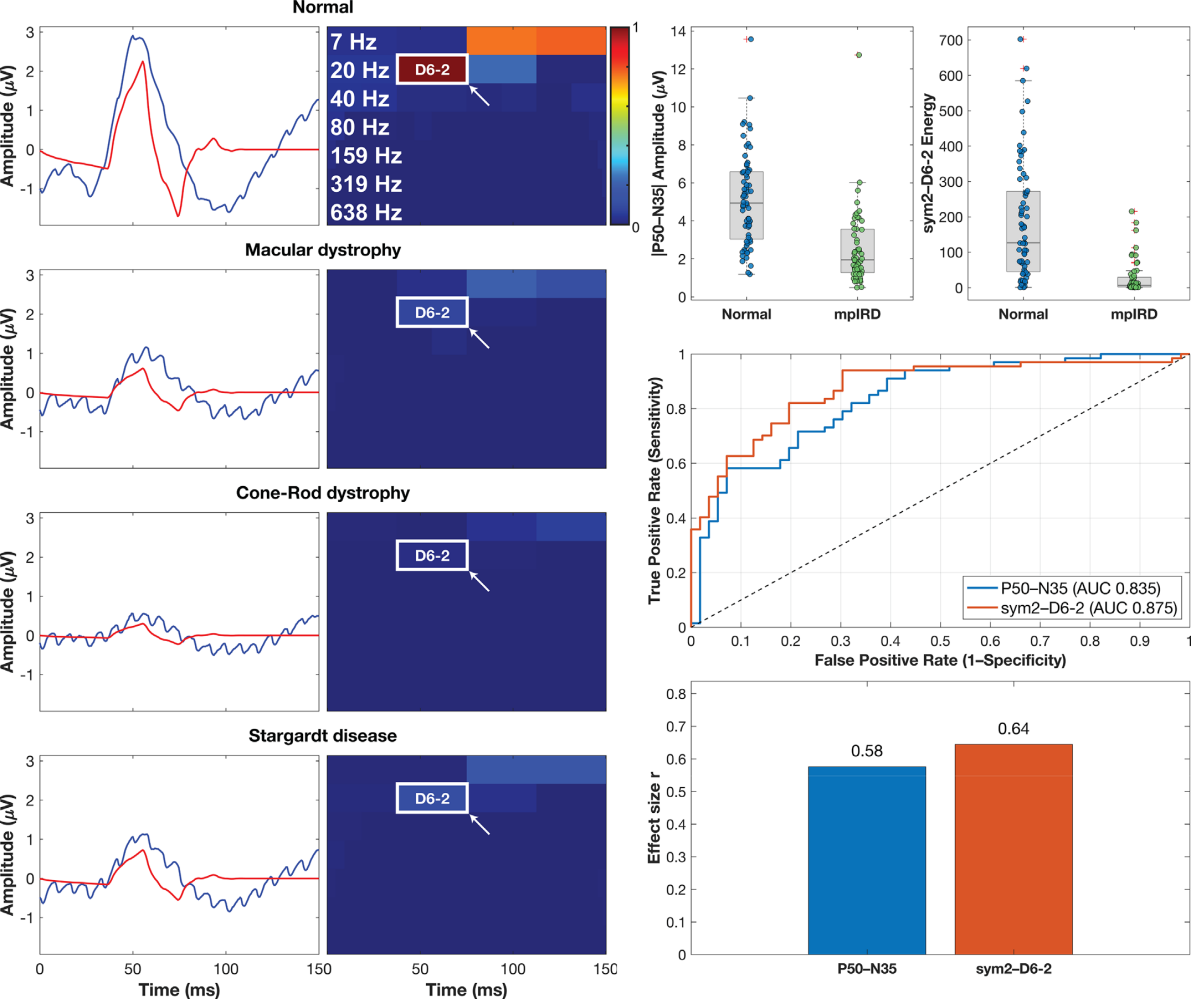
Comparative diagnostic performance of the wavelet feature sym2–D6-2 versus the conventional amplitude metric |P50–N35|. *Left column:* Averaged PERG recordings (*blue*) with reconstructed signals using only sym2–D6-2 (*red*) and corresponding sym2 scalograms for normal, MD, CRD, and STGD recordings (*white box* and *arrow* highlighting the D6-2 index). *Right column:* *Top*: Tukey boxplots show markedly lower |P50–N35| amplitudes and sym2–D6-2 energies in mpIRDs versus normals; *circles* denote individual participants. *Middle*: ROC curves demonstrate greater discriminative ability for sym2–D6-2 (AUC = 0.875) than |P50–N35| (AUC = 0.835). *Bottom*: Effect size bars (Mann–Whitney *r*) confirm the superiority of sym2–D6-2 (*r_es_* = 0.644) over |P50–N35| (*r_es_* = 0.576). Together, the analysis reinforces sym2–D6-2 as a sensitive marker of macular cone dysfunction. *r_es_*: effect size.

Lastly, we obtained the averaged time-domain plot (blue) and scalograms of all normal recordings and participants with MD, CRD, and STGD in Figure 5, with signal reconstruction (red plot line) using only the sym2–D6-2 index (white outline scalogram). Tukey boxplots demonstrate large group differences between normal participants and mpIRDs for both |P50–N35| and sym2–D6-2, with sym2–D6-2 yielding nonoverlapping quartile boxes. ROC analysis reinforces these findings, with sym2–D6-2 yielding an AUC of 0.875, outperforming |P50–N35| (AUC = 0.835).

## Discussion

To our knowledge, this is the first study to analyze the PERG-IOBA data set, as well as the first to assess macular cone time-frequency features in PERG recordings. We successfully applied a robust DWT analysis of PERG recordings in normal participants and those with mpIRDs to elucidate novel time-frequency markers. We utilized the DWT as it produces orthogonal, nonredundant coefficient indices that make statistical testing and physiological mapping straightforward. Other similar methods also include the continuous wavelet transform (CWT) and variable-frequency complex demodulation (VFCDM).^35^ Although the CWT offers finer frequency resolution by evaluating every scale and shift, it is redundant, blurs timing accuracy for short events, and is computationally expensive. Alternatively, VFCDM offers an adaptive high-resolution spectrogram that excels at tracking slowly drifting rhythms. However, it lacks an inverse transform that directly reconstructs individual peaks such as N35, P50, or N95 in the PERG. Nonetheless, both methods may enrich machine learning (ML) approaches for diagnostic classification and will be considered in future work.

Previous work has applied DWT to PERG recordings, although with different aims.^20^^,^^28^ A study by Rogala and Brykalski^20^ showed that DWT coefficients improved the separation of healthy and diseased PERG recordings over peak-based features. Using principal component analysis to visualize the feature space and the k-means clustering algorithm for classification, they reported lower misclassification rates for wavelet-based features (34% and 36%) compared to conventional peak parameters (60% and 55%). A later study on patients with early primary open-angle glaucoma (POAG) by Hassankarimi et al.^28^ systematically compared multiple mother wavelets and decomposition levels across small-check (0.8°) and large-check (16°) stimuli, evaluating features such as mean, standard deviation, and relative energy. They found that with the Daubechies 8 (db8) mother wavelet, the relative energy of the level 7 third coefficient, termed “7N,” in small-check stimuli was the only measure to significantly discriminate early POAG from controls. Both studies demonstrated DWT’s superior utility in analyzing PERG recordings, but neither sought to specifically analyze a macular cone time-frequency feature. The present work does exactly that, by systematically aligning wavelet-derived indices with canonical PERG components and utilizing an energy-to-entropy screen to identify physiologically grounded markers of macular cone function.

We first performed energy-to-entropy analysis of 20 candidate mother wavelets to maximize signal fidelity (high energy) while minimizing noise or signal distortion imparted by transforms (low entropy). Of the final selected wavelets, sym2 emerged as the top-performing index for our aim (2). Its symmetric design and two vanishing moments are an excellent balance of reduced phase distortion and high spectral resolution. Furthermore, because the time-domain shape of the sym2 wavelet resembles PERG peaks (N35, P50, N95), the convolution produces larger coefficients, maximizing energy compaction at clinically relevant components. For similar reasons, we also used sym2 as our mother wavelet of choice in aim (3). Other studies have conducted energy-to-entropy analysis of ERG recordings,^19^^,^^27^ with Sarossy et al.^27^ finding coif8 to yield the highest energy-to-entropy ratio. However, their study sampled recordings at 4000 Hz, whereas the data utilized here were sampled at 1700 Hz and placed through a 1- to 100-Hz bandpass filter. Given our narrower frequency range, higher-order wavelets were less suited for our observed signals. Additionally, higher-order wavelets like coif8 exhibit longer filter lengths and many vanishing moments, likely introducing boundary artifacts and excessive smoothing of transient peaks. The study by Gao et al.^19^ on oscillatory potentials (OPs) in light-adapted ERGs exhibited a similar trend to our work, where lower-order wavelets yielded higher ratios, with sym2 having the highest ratio.

Inspiration for aim (2) stemmed from previous work by Gauvin and colleagues,^26^^,^^29^^,^^36^ where they first identified DWT indices indicative of ON- and OFF-pathway responses.^29^ By assessing ffERG recordings in patients with ON-pathway loss (type 1 congenital stationary night blindness) and OFF-pathway loss (congenital postreceptoral cone pathway anomaly), their studies showed that transient energy indices in the 20-Hz and 40-Hz bands represent ON- and OFF-pathway activity, respectively. Furthermore, they demonstrated that the 40:20 ratio flips polarity exactly as pathway dominance reverses. This patient-centric approach converted abstract DWT indices into clinically meaningful descriptors of retinal circuitry. We applied the same approach to our assessment of healthy controls and those with mpIRDs. These disorders selectively disrupt macular cone function, ultimately resulting in loss of a P50 response in PERG recordings, providing a natural test population for descriptors that quantify macular cone portions. Among the 141 features we screened, sym2–D6-2, a time-frequency descriptor spanning 27 to 53 Hz and 38 to 75 ms, the temporal range containing P50, emerged as the strongest discriminator (*r_es_* = 0.644, CLES = 0.875) between healthy and mpIRD eyes. We also assessed the correlation of |P50–N35|, a quantitative marker of macular cone activity,^5^ with sym2–D6-2 across healthy controls in Figure 4 yielding a high correlation (*r_corr_* = 0.95). Additionally, Figure 5 visually demonstrates sym2–D6-2’s ability to largely capture the positive P50 inflection seen in PERG recordings. Tukey boxplots in Figure 5 demonstrate sym2–D6-2 is better able to delineate pathology versus normal recordings as they are nonoverlapping, unlike |P50–N35|. Ultimately, the biological concordance, disease specificity, temporal alignment with P50 macular cone activity, and statistical primacy strongly suggest sym2–D6-2 as a compact, quantitative index of macular cone function.

Previous work^19^^,^^22^ has also demonstrated the need for multiple DWT indices to fully represent selective portions of light-adapted OPs. For example, Gauvin et al.^22^ found two decomposition levels, at 80 Hz and 160 Hz, to contain indices selectively representative of OPs. Here, our second highest-performing feature, coif1–D5-3 (38–56 ms, 27–53 Hz, *r_es_* = 0.621, CLES = 0.861), spans a different portion of the same macular cone window (N35 → P50) while also occupying a different frequency band. The selective high performance of this wavelet at specific times and frequencies reflects its inherent strengths. Specifically, coif1 is well suited for resolving sharp peaks—fittingly, coif1–D5-3 (38–56 ms) encompasses the P50 peak. Leveraging the strengths of each mother wavelet at distinct time and frequency portions of the macular cone response may yield a minimal set of features that are highly representative of macular cone response dynamics (transient high-frequency responses captured by haar, large-slow waveform dynamics by sym2, and high peak resolution by coif1).

Sym2–D6-3 (75–112 ms, 13–27 Hz) is the fourth highest-ranked feature (*r_es_* = 0.581; CLES = 0.838) and temporally encompasses the N95 component. Previous work has demonstrated how advanced loss of retinal cells results in secondary loss of neurosensory function, including RGCs.^37^^,^^38^ Although it is plausible sym2–D6-3 captures RGC activity, further validation with a curated cohort exhibiting primary RGC dysfunction may be better suited to confirm this. Lastly, fk4–D6-2 demonstrates the utility of energy-to-entropy analysis, with it yielding a lower ratio for D6, as shown in Figure 2. It represents the same time-frequency range as our highest-ranked feature, sym2–D6-2, yet exhibits a lower effect size, likely due to additional noise and distortion imparted by the fk4 mother wavelet. Furthermore, it was not removed from analysis due to redundancy, as it produced a |ρ| value of 0.89, just below the studies’ 0.9 cutoff.

The algorithmic, minimal coefficient signal reconstruction approach aim (3) provides an independent line of evidence that converges on the same dominant time-frequency descriptors highlighted by our statistical screen aim (2). Isolating individual bands and then iteratively resynthesizing the signal showed that three detailed (sym2–D5-2, sym2–D6-2, sym2–D6-3) and two approximation coefficients (sym2–A6-3, sym2–A6-4) were collectively sufficient to minimally reconstruct canonical portions of the PERG waveform with high fidelity. Two of these coefficients are the very ones that emerged as top performers in our statistical assessment of features, as they reproducibly mapped onto canonical clinical intervals: sym2–D6-2 aligning with |P50–N35| and sym2–D6-3 with |N95–P50|. The agreement between statistical ranking and minimal algorithmic signal reconstruction strengthens the biological plausibility of these descriptors, notably sym2–D6-2, which spans ∼38 to 75 ms and coincides temporally with the P50 component. The sym2 mother wavelet exhibits a temporal profile closely matching the macular cone response, making it more likely to be captured by DWT convolutions.^39^ The D6 frequency band (13–27 Hz) corresponds to lower-frequency regions in which cellular responses to the PERG checkerboard reversal are expected, given PERG's dominance by RGC cell bodies and axons.^40^^,^^41^ Within this band, the −2 index captures activity spanning ∼38 to 75 ms, overlapping temporally with the P50 component, being a well-established measure of macular cone integrity.^5^^,^^42^ Together, these properties support the interpretation of sym2–D6-2 as a macular cone–driven feature. Moreover, because each index captures energy over a defined time-frequency window, these DWT indices could serve as robust surrogates for macular cone function in situations where PERG profiles are flattened and conventional amplitude/implicit-time measurements are difficult to obtain.

Although the findings of this study are promising, a few limitations should be acknowledged. First, the data set utilized here is limited in that it only contains demographic information, VA measures, and three ophthalmologist-confirmed diagnoses. Established quantitative imaging and structural markers such as those from fundus autofluorescence and optical coherence tomography could yield a deeper context for interpreting time-frequency domains identified in this study. Additionally, the current analysis does not take advantage of any longitudinal data. Future studies that incorporate repeated recordings from the same individuals alongside these advanced imaging modalities would allow us to better evaluate whether these wavelet indices can serve as reliable biomarkers in clinical trials and potentially help guide patient management.

Additionally, recent work has demonstrated that ML wrappers, coupled with explainable artificial intelligence, can provide complementary insights.^43^^–^^45^ For example, tree-based ensembles like random forests and XGBoost trained on a joint set of DWT energies, along with canonical time-domain metrics, yield per-feature importance via Shapley Additive exPlanations (SHAP).^46^ Compared to univariate ranking, SHAP can uncover higher-order interactions, account for marginal contributions after accounting for collinearity, and highlight causes for misclassification. However, this method typically requires cross-validation to avoid overfitting, which was not available for the current study. Subsequent investigations will look to acquire an external validation data set that will enable these alternative feature extraction methods, as well as the development of an ML classifier. Other considerations include integration with semantic ontologies to better uncover genotype–phenotype associations, improve diagnostic precision, and facilitate data interoperability.^47^

In conclusion, our results demonstrate that implementing DWT in the analysis of PERG yields a multidimensional, reproducible, quantitative means to represent canonical PERG markers. We have demonstrated that sym2–D6-2 captures macular cone activity through a selective cohort design, statistical approach, and minimal algorithmic signal reconstruction. These time-frequency features can serve as objective markers of function, as they remain directly quantifiable and well suited for longitudinal monitoring where signal quality or response amplitude may decline with disease progression.

## Supplementary Material

Supplement 1

Supplement 2

## References

[1] Yu M, Creel D, Iannaccone A, eds. *Handbook of Clinical Electrophysiology of Vision*. Switzerland: Springer Nature; 2021.

[2] McAnany JJ, Persidina OS, Park JC. Clinical electroretinography in diabetic retinopathy: a review. *Surv Ophthalmol*. 2022; 67(3): 712–722. 34487740 10.1016/j.survophthal.2021.08.011 PMC9158180

[3] Holder GE . Pattern electroretinography (PERG) and an integrated approach to visual pathway diagnosis. *Prog Retin Eye Res*. 2001; 20(4): 531–561. 11390258 10.1016/s1350-9462(00)00030-6

[4] Holder GE . The pattern electroretinogram in anterior visual pathway dysfunction and its relationship to the pattern visual evoked potential: a personal clinical review of 743 eyes. *EYE*. 1997; 11(pt 6): 924–934. 9537157 10.1038/eye.1997.231

[5] Thompson DA, Bach M, McAnany JJ, Šuštar Habjan M, Viswanathan S, Robson AG. ISCEV standard for clinical pattern electroretinography (2024 update). *Doc Ophthalmol*. 2024; 148(2): 75–85. 38488946 10.1007/s10633-024-09970-1 PMC10954931

[6] Ratra D, Ozdek S, Raviselvan M, Elchuri S, Sharma T. Approach to inherited retinal diseases. *Indian J Ophthalmol*. 2022; 70(7): 2305–2315. 35791111 10.4103/ijo.IJO_314_22 PMC9426075

[7] Hamel CP . Cone rod dystrophies. *Orphanet J Rare Dis*. 2007; 2(1): 7. 17270046 10.1186/1750-1172-2-7 PMC1808442

[8] Downes SM, Holder GE, Fitzke FW, et al. Autosomal dominant cone and cone-rod dystrophy with mutations in the guanylate cyclase activator 1A gene-encoding guanylate cyclase activating protein-1. *Arch Ophthalmol*. 2001; 119(1): 96–105. 11146732

[9] Lois N, Holder GE, Bunce C, Fitzke FW, Bird AC. Phenotypic subtypes of Stargardt macular dystrophy-fundus flavimaculatus. *Arch Ophthalmol*. 2001; 119(3): 359–369. 11231769 10.1001/archopht.119.3.359

[10] Broadgate S, Yu J, Downes SM, Halford S. Unravelling the genetics of inherited retinal dystrophies: past, present and future. *Prog Retin Eye Res*. 2017; 59: 53–96. 28363849 10.1016/j.preteyeres.2017.03.003

[11] Lam BL, Leroy BP, Black G, Ong T, Yoon D, Trzupek K. Genetic testing and diagnosis of inherited retinal diseases. *Orphanet J Rare Dis*. 2021; 16(1): 514. 34906171 10.1186/s13023-021-02145-0 PMC8670140

[12] Duncan JL, Branham K, Birch DG, et al. Guidelines on clinical assessment of patients with inherited retinal degenerations. *American Academy of Ophthalmology*. 2022. Accessed September 4, 2025, https://www.aao.org/education/clinical-statement/guidelines-onclinical-assessment-of-patients-with.

[13] Yang TH, Kang EYC, Lin PH, Wu PL, Sachs JA, Wang NK. The value of electroretinography in identifying candidate genes for inherited retinal dystrophies: a diagnostic guide. *Diagnostics (Basel)*. 2023; 13(19): 3041. 37835784 10.3390/diagnostics13193041 PMC10572658

[14] Lenassi E, Jarc-Vidmar M, Glavac D, Hawlina M. Pattern electroretinography of larger stimulus field size and spectral-domain optical coherence tomography in patients with Stargardt disease. *Br J Ophthalmol*. 2009; 93(12): 1600–1605. 19628494 10.1136/bjo.2009.158725

[15] Sajovic J, Meglič A, Hawlina M, Fakin A. Electroretinography as a biomarker to monitor the progression of Stargardt disease. *Int J Mol Sci*. 2022; 23(24): 16161. 36555803 10.3390/ijms232416161 PMC9783580

[16] Fujinami K, Lois N, Davidson AE, et al. A longitudinal study of Stargardt disease: clinical and electrophysiologic assessment, progression, and genotype correlations. *Am J Ophthalmol*. 2013; 155(6): 1075–1088.e13. 23499370 10.1016/j.ajo.2013.01.018

[17] Hadalin V, Šuštar M, Volk M, et al. Cone dystrophy associated with a novel variant in the terminal codon of the RPGR-ORF15. *Genes (Basel)*. 2021; 12(4): 499. 33805381 10.3390/genes12040499 PMC8066792

[18] Michaelides M, Holder GE, Webster AR, et al. A detailed phenotypic study of “cone dystrophy with supernormal rod ERG.” *Br J Ophthalmol*. 2005; 89(3): 332–339. 15722315 10.1136/bjo.2004.050567 PMC1772537

[19] Gao M, Barboni MTS, Szabó V, Nagy ZZ, Zobor D, Nagy BV. Oscillatory potential-based characterization of the human light-adapted electroretinogram using discrete wavelet transform. *Period Polytech Mech Eng*. 2024; 68(3): 187–195.

[20] Rogala T, Brykalski A. Wavelet feature space in computer-aided electroretinogram evaluation. *Pattern Anal Appl*. 2005; 8(3): 238–246.

[21] Gauvin M, Chakor H, Koenekoop RK, Little JM, Lina JM, Lachapelle P. Witnessing the first sign of retinitis pigmentosa onset in the allegedly normal eye of a case of unilateral RP: a 30-year follow-up. *Doc Ophthalmol*. 2016; 132(3): 213–229. 27041556 10.1007/s10633-016-9537-y

[22] Gauvin M, Dorfman AL, Trang N, et al. Assessing the contribution of the oscillatory potentials to the genesis of the photopic ERG with the discrete wavelet transform. *Biomed Res Int*. 2016; 2016: 2790194. 28101507 10.1155/2016/2790194 PMC5217158

[23] Dimopoulos IS, Freund PR, Redel T, Dornstauder B, Gilmour G, Sauvé Y. Changes in rod and cone-driven oscillatory potentials in the aging human retina. *Invest Ophthalmol Vis Sci*. 2014; 55(8): 5058–5073. 25034601 10.1167/iovs.14-14219

[24] Kundra H, Park JC, McAnany JJ. Comparison of photopic negative response measurements in the time and time-frequency domains. *Doc Ophthalmol*. 2016; 133(2): 91–98. 27562839 10.1007/s10633-016-9558-6 PMC5053889

[25] Zhdanov A, Constable P, Manjur SM, Dolganov A, Posada-Quintero HF, Lizunov A. OculusGraphy: signal analysis of the electroretinogram in a rabbit model of endophthalmitis using discrete and continuous wavelet transforms. *Bioengineering (Basel)*. 2023; 10(6): 708. 37370639 10.3390/bioengineering10060708 PMC10294831

[26] Gauvin M, Lina JM, Lachapelle P. Advance in ERG analysis: from peak time and amplitude to frequency, power, and energy. *Biomed Res Int*. 2014; 2014: 246096. 25061605 10.1155/2014/246096 PMC4100345

[27] Sarossy M, Crowston J, Kumar D, Weymouth A, Wu Z. Time-frequency analysis of ERG with discrete wavelet transform and matching pursuits for glaucoma. *Transl Vis Sci Technol*. 2022; 11(10): 19. 10.1167/tvst.11.10.19 PMC9583752 36227605

[28] Hassankarimi H, Noori SMR, Jafarzadehpour E, Yazdani S, Radinmehr F. Analysis of pattern electroretinogram signals of early primary open-angle glaucoma in discrete wavelet transform coefficients domain. *Int Ophthalmol*. 2019; 39(10): 2373–2383. 30725244 10.1007/s10792-019-01077-w

[29] Gauvin M, Sustar M, Little JM, Brecelj J, Lina JM, Lachapelle P. Quantifying the ON and OFF contributions to the flash ERG with the discrete wavelet transform. *Transl Vis Sci Technol*. 2017; 6(1): 3. 10.1167/tvst.6.1.3 PMC5235331 28097047

[30] Fernández I, Cuadrado-Asensio R, Larriba Y, Rueda C, Coco-Martín RM. A comprehensive dataset of pattern electroretinograms for ocular electrophysiology research. *Sci Data*. 2024; 11(1): 1013. 39294170 10.1038/s41597-024-03857-1 PMC11410942

[31] Goldberger AL, Amaral LA, Glass L, et al. PhysioBank, PhysioToolkit, and PhysioNet: components of a new research resource for complex physiologic signals. *Circulation*. 2000; 101(23): E215–20. 10851218 10.1161/01.cir.101.23.e215

[32] Bach M, Brigell MG, Hawlina M, et al. ISCEV standard for clinical pattern electroretinography (PERG): 2012 update. *Doc Ophthalmol*. 2013; 126(1): 1–7. 23073702 10.1007/s10633-012-9353-y

[33] Shwetar Y, Lalush D, McAnany J, Jeffrey B, Haendel M. A practical introduction to wavelet analysis in electroretinography. *medRxiv*. Published online July 27, 2025, doi:10.1101/2025.07.25.25331915. PMC12948838 41398515

[34] McGraw KO, Wong SP. A common language effect size statistic. *Psychol Bull*. 1992; 111(2): 361–365.

[35] Behbahani S, Ahmadieh H, Rajan S. Feature extraction methods for electroretinogram signal analysis: a review. *IEEE Access*. 2021; 9: 116879–116897.

[36] Gauvin M, Little JM, Lina JM, Lachapelle P. Functional decomposition of the human ERG based on the discrete wavelet transform. *J Vis*. 2015; 15(16): 14. 10.1167/15.16.14 26746684

[37] Marc RE, Jones BW, Watt CB, Strettoi E. Neural remodeling in retinal degeneration. *Prog Retin Eye Res*. 2003; 22(5): 607–655. 12892644 10.1016/s1350-9462(03)00039-9

[38] Garcia-Ayuso D, Di Pierdomenico J, Agudo-Barriuso M, Vidal-Sanz M, Villegas-Pérez MP. Retinal remodeling following photoreceptor degeneration causes retinal ganglion cell death. *Neural Regen Res*. 2018; 13(11): 1885–1886. 30233058 10.4103/1673-5374.239436 PMC6183041

[39] Marxim Rahula Bharathi B, Balaji NS, Meena R, et al. Unveiling optimal mother wavelets by COPRAS method analyzing speech signals despite face mask and shield obstacles. *Sci Rep*. 2025; 15(1): 14044. 40269098 10.1038/s41598-025-97823-5 PMC12019381

[40] Luo X, Frishman LJ. Retinal pathway origins of the pattern electroretinogram (PERG). *Invest Ophthalmol Vis Sci*. 2011; 52(12): 8571–8584. 21948546 10.1167/iovs.11-8376 PMC3208386

[41] Ventura LM, Sorokac N, De Los Santos R, Feuer WJ, Porciatti V. The relationship between retinal ganglion cell function and retinal nerve fiber thickness in early glaucoma. *Invest Ophthalmol Vis Sci*. 2006; 47(9): 3904–3911. 16936103 10.1167/iovs.06-0161 PMC1808329

[42] Calcagni A, Neveu MM, Jurkute N, Robson AG. Electrodiagnostic tests of the visual pathway and applications in neuro-ophthalmology. *EYE*. 2024; 38(12): 2392–2405. 38862643 10.1038/s41433-024-03154-6 PMC11306601

[43] Barredo Arrieta A, Díaz-Rodríguez N, Del Ser J, et al. Explainable artificial intelligence (XAI): concepts, taxonomies, opportunities and challenges toward responsible AI. *Inf Fusion*. 2020; 58: 82–115.

[44] Solomon BD, Cheatham M, de Guimarães TAC, et al. Perspectives on the current and future state of artificial intelligence in medical genetics [published online May 15, 2025]. *Am J Med Genet A*, 10.1002/ajmg.a.64118. PMC13255970 40375359

[45] Shwetar YJ, Brooks BP, Jeffrey BG, Solomon BD, Haendel MA. Advances in machine learning for ABCA4-related retinopathy: segmentation and phenotyping. *Int Ophthalmol*. 2025; 45(1): 314. 40699379 10.1007/s10792-025-03690-4 PMC12287237

[46] Constable PA, Pinzon-Arenas JO, Mercado Diaz LR, et al. Spectral analysis of light-adapted electroretinograms in neurodevelopmental disorders: Classification with machine learning. *Bioengineering (Basel)*. 2024; 12(1): 15. 39851292 10.3390/bioengineering12010015 PMC11761560

[47] Shwetar Y, Toro S, Matentzoglu N, et al. Advancing the Mondo disease ontology to improve the precision diagnosis and care of eye diseases. *Invest Ophthalmol Vis Sci*. 2025; 66(8): 2726–2726.

