# Supplementary material for: Multidimensional Quantification of Macular Cone Activity in Pattern Electroretinography Using Discrete Wavelet Transform
Source: Transl Vis Sci Technol. 2025 Sep 12;14(9):17. doi: 10.1167/tvst.14.9.17 (PMC12439505; doi:10.1167/tvst.14.9.17)
Supplement: Supplement 1 [file tvst-14-9-17_s001.docx]

| **Supplemental Table 1: Final Demographics Diagnosis Labels** | | | | |
| --- | --- | --- | --- | --- |
| Subjects | Recordings | diagnosis1 | diagnosis2 | diagnosis3 |
| 67 | 262 | "Normal" | "" | "" |
| 13 | 56 | "Stargardt disease" | "" | "" |
| 11 | 42 | "Cone-Rod dystrophy" | "" | "" |
| 6 | 28 | "Macular dystrophy" | "Central areolar choroidal dystrophy" | "" |
| 6 | 24 | "Macular dystrophy" | "Stargardt disease" | "Fundus flavimaculatus" |
| 5 | 18 | "Macular dystrophy" | "Periferina" | "" |
| 4 | 14 | "Macular dystrophy" | "Vitelliform macular dystrophy" | "" |
| 3 | 10 | "Macular dystrophy" | "Dominant drusen" | "" |
| 2 | 8 | "Macular dystrophy" | "" | "" |
| 1 | 4 | "Cone-Rod dystrophy" | "Fundus flavimaculatus" | "" |
| 1 | 4 | "Macular dystrophy" | "Alport syndrome: Systemic disorder with ocular manifestations" | "" |
| 1 | 4 | "Macular dystrophy" | "Benign concentric annular macular dystrophy" | "" |
| 1 | 4 | "Macular dystrophy" | "Sorsby Fundus Dystrophy" | "" |
| 1 | 4 | "Stargardt disease" | "Cone-Rod dystrophy" | "" |
| 1 | 4 | "Stargardt disease" | "Fundus flavimaculatus" | "" |


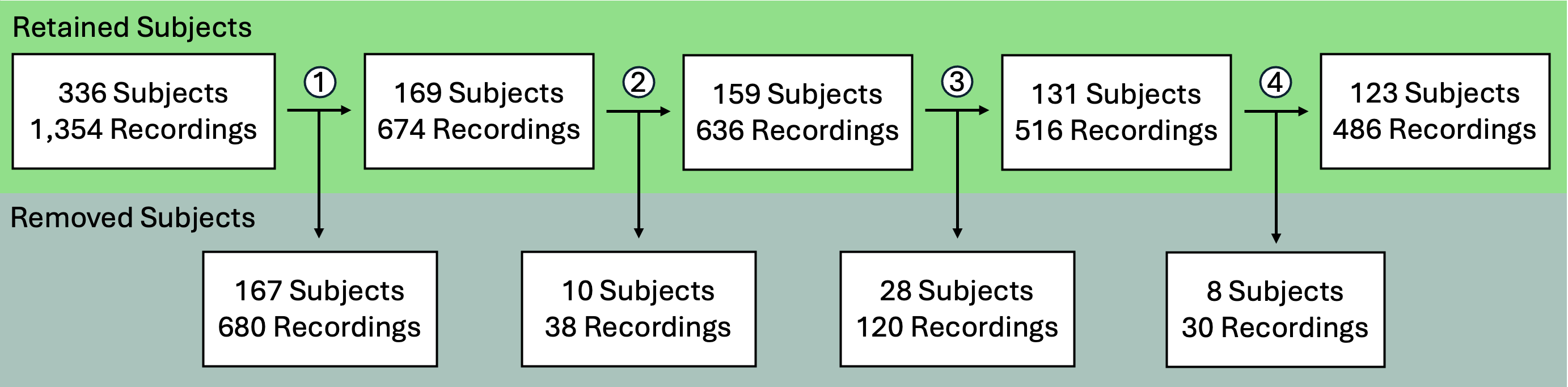
**Supplemental Figure 1: Process of retaining and removing subjects and recordings.** (1) Retained subjects/recordings with “Normal”, “Stargardt disease”, “Cone-Rod dystrophy”, or “Macular dystrophy” in the *diagnosis1* column. (2) Retained subjects/recordings with a visual acuity (VA) measure. (3) Retained subjects/recordings without “Mercury poisoning” in the *comments* column. (4) Retained recordings from a subjects first visit only.

Many subjects in the final dataset (as outlined above) carried more than one diagnosis (diagnosis2 or diagnosis3 columns in Supplemental Table 1). In most cases, these secondary labels represent alternate nomenclature for the primary macular condition or related phenotypes with similar cone–rod or macular involvement and are not expected to introduce unrelated retinal physiology.

Central areolar choroidal dystrophy is a late-onset macular dystrophy primarily involving cone photoreceptors, producing functional loss like that seen in the “Macular dystrophy” primary diagnosis.

Fundus flavimaculatus is a phenotypic variation of ABCA4-associated Stargardt disease, sharing the same underlying pathophysiology.

Peripherina (“Periferina” as provided in the dataset) refers to mutations in the PRPH2 gene, which can present with macular dystrophy phenotypes and typically spares the peripheral retina in early disease.

Vitelliform macular dystrophy (Best disease) and Dominant drusen are both localized macular disorders affecting the retinal pigment epithelium/photoreceptor interface.

Alport syndrome is a systemic basement membrane disorder that can include retinal changes such as dot-and-fleck retinopathy but generally does not contribute to isolated electroretinographic deficits beyond those attributable to the coexisting macular dystrophy diagnosis.

Benign concentric annular macular dystrophy is a rare macular dystrophy with central cone involvement, with expected PERG changes like other macular dystrophies.

Sorsby fundus dystrophy is a late-onset macular degeneration caused by TIMP3 mutations; while advanced disease may include choroidal neovascularization, early disease remains macular-predominant.

As demonstrated above, the secondary and tertiary diagnoses in this dataset are either alternate descriptors, closely related phenotypes, or additional macular-limited disorders that are unlikely to substantially influence the interpretation of PERG-derived time–frequency features.
