# Supplementary material for: Multidimensional Quantification of Macular Cone Activity in Pattern Electroretinography Using Discrete Wavelet Transform
Source: Transl Vis Sci Technol. 2025 Sep 12;14(9):17. doi: 10.1167/tvst.14.9.17 (PMC12439505; doi:10.1167/tvst.14.9.17)
Supplement: Supplement 2 [file tvst-14-9-17_s002.docx]

**SUPPLEMENTARY MATERIAL**

| **Table S2. Decomposition Level Frequency Ranges and Center Frequencies** | |
| --- | --- |
| Decomposition Level | Frequency Band (Hz) (center) |
| D1 | 425.0 – 850.0 (637.5) |
| D2 | 212.5 – 425.0 (318.8) |
| D3 | 106.2 – 212.5 (159.4) |
| D4 | 53.1 – 106.2 (79.7) |
| D5 | 26.6 – 53.1 (40.0) |
| D6 | 13.3 – 26.6 (19.9) |
| D7 | 6.6 – 13.3 (10.0) |
| A5 | 0 – 26.6 (13.3) |
| A6 | 0 – 13.3 (6.6) |
| A7 | 0 – 6.6 (3.3) |

D1-D7, A7: haar

D1-D6, A6: sym2 and fk4

D1-D5, A5: sym4, db4, coif1

| **Table S3: Top Features (LE Only)** | | | | |
| --- | --- | --- | --- | --- |
| Feature | Effect size *r* | CLES | Time range (ms) | Frequency range (Hz) |
| sym2-D6-2 | 0.606 | 0.853 | 13 – 27 | 38 – 75 |
| sym4-D5-3 | 0.582 | 0.839 | 27 – 53 | 38 – 56 |
| fk4-D4-5 | 0.559 | 0.825 | 53 – 106 | 38 – 46 |
| sym2-D6-3 | 0.557 | 0.824 | 13 – 27 | 75 – 112 |
| fk4-D6-2 | 0.538 | 0.813 | 13 – 27 | 38 – 75 |
| coif1-A5-4 | 0.521 | 0.803 | 0 – 27 | 56 – 75 |

| **Table S4: Top Features (RE Only)** | | | | |
| --- | --- | --- | --- | --- |
| Feature | Effect size *r* | CLES | Time range (ms) | Frequency range (Hz) |
| haar-A7-2* | 0.601 | 0.850 | 75 – 150 | 0 – 7 |
| coif1-D5-3 | 0.592 | 0.844 | 38 – 56 | 27 – 53 |
| coif1-A5-4 | 0.575 | 0.835 | 56 – 75 | 0 – 27 |
| coif1-D5-2 | 0.557 | 0.824 | 19 – 37 | 27 – 53 |
| sym2-D6-3 | 0.551 | 0.821 | 75 – 112 | 13 – 27 |
| fk4-D6-2 | 0.549 | 0.820 | 38 – 75 | 13 – 27 |

| **Table S5: Top Features (Lower VA Only)** | | | | |
| --- | --- | --- | --- | --- |
| Feature | Effect size *r* | CLES | Time range (ms) | Frequency range (Hz) |
| haar-A7-2* | 0.589 | 0.843 | 75 – 150 | 0 – 7 |
| sym4-A5-3 | 0.569 | 0.831 | 38 – 56 | 0 – 27 |
| coif1-D5-3 | 0.568 | 0.830 | 38 – 56 | 27 – 53 |
| sym2-D6-3 | 0.564 | 0.828 | 75 –112 | 13 – 27 |
| coif1-D5-2 | 0.530 | 0.808 | 19 – 37 | 27 – 53 |
| haar-D4-5 | 0.510 | 0.800 | 38 – 46 | 53 – 106 |

| **Table S6: Top Features (Lower \|P50 - N35\| Only)** | | | | |
| --- | --- | --- | --- | --- |
| Feature | Effect size *r* | CLES | Time range (ms) | Frequency range (Hz) |
| sym4-D5-3 | 0.536 | 0.812 | 27 – 53 | 38 – 56 |
| haar-A7-2* | 0.514 | 0.800 | 0 – 7 | 75 – 150 |
| sym2-D6-3 | 0.493 | 0.787 | 13 – 27 | 75 – 112 |
| db4-D5-3 | 0.483 | 0.781 | 27 – 53 | 38 – 56 |
| sym4-A5-3 | 0.482 | 0.780 | 0 – 27 | 38 – 56 |
| fk4-D6-2 | 0.465 | 0.771 | 13 – 27 | 38 – 75 |

*sym2-D6-2 was pruned from Tables S4 (RE), S5 (lower VA), and S6 (lower |P50 - N35|) due to high correlation (|ρ| > 0.9) with haar-A7-2. Haar-A7-2 temporally (75–150 ms) and spectrally (0–7 Hz) aligns with retinal ganglion cell/optic nerve firing linked to the stimulus reversal rate. Given upstream macular cone loss, a corresponding downstream reduction in this biological response is expected.
